# Evaluation of Oral Health Status Using the Geriatric Oral Health Assessment Index Among the Geriatric Population in India: A Pilot Study

**DOI:** 10.7759/cureus.7344

**Published:** 2020-03-20

**Authors:** Akshaya Venkatesan, Annie Sylvea V, Suganya Ramalingam, Madhan Kumar Seenivasan, Malathi Narasimhan

**Affiliations:** 1 Oral Pathology, Faculty of Dental Sciences, Sri Ramachandra Institution of Higher Education and Research, Chennai, IND; 2 Oral Pathology, Faculty of Dental Sciences, Sri Ramachandra Institute of Higher Education and Research, Chennai, IND; 3 Oral Pathology, Sri Ramachandra Institute of Higher Education and Research, Chennai, IND; 4 Prosthodontics, Sri Ramachandra Institute of Higher Education and Research, Chennai, IND

**Keywords:** geriatric oral health assessment index, geriatric, oral health, assessment, xerostomia, swallowing

## Abstract

Introduction and aim

Clinical indicators alone are insufficient for evaluating oral health. In addition to health and disease, oral health includes socio-dental indicators of physical, psychological, and social aspects of well-being. The adaptive capacity of an individual influences the perception of oral health-related quality of life (OHRQoL). Indices such as the Oral Health Impact Profile, Oral Impacts on Daily Performances, and the Geriatric Oral Health Assessment Index (GOHAI) have been used to measure OHRQoL. This study was designed to assess OHRQoL in older individuals using the GOHAI.

Methods

Subjects aged older than 65 years who visited our institution from January to March 2016 were included. Subjects with cognitive behavior disorders were excluded. Subjects were assigned into three groups based on age: 65-69 years, 70-74 years, and 75 years or older. The participants were asked 12 questions, and their responses were assessed by age group. Our Institutional Ethics Committee approved the study protocol.

Results

The 219 subjects recruited included 126 (57.5%) patients aged 65-69 years, 57 (26.0%) patients aged 70-74 years, and 36 (16.4%) patients aged 75 years or older. Several physical, physiological, and psychological aspects of the GOHAI differed significantly among these three groups, with overall OHRQoL decreasing with age.

Conclusion

Although oral healthcare problems were widespread in the geriatric population, they were not a primary concern. Attitudes toward dentistry require improvement. However, further studies in larger populations are required to assess geriatric oral health.

## Introduction

Estimates indicate that, by 2050, 17% of the Indian population will be aged 65 years or older, compared with 7.4% in that age range in 2001 [1]. The number of older adults needing dental care is increasing, as is the need to provide these subjects with optimum dental care. Age-related deterioration in oral health adversely affects the physical and psychological health of individuals, decreasing their overall quality of life (QoL) [2]. Providing optimum dental care requires the assessment of oral health and its impact on QoL. One of the most specific methods of evaluating oral health in older subjects and its impact on their QoL is the Geriatric Oral Health Assessment Index (GOHAI), which was developed to assess oral health status in geriatric subjects [3-4]. This index has shown satisfactory internal consistency, and its validity has been confirmed in many international studies [5]. The GOHAI has been used to evaluate the physical, physiological, and psychological aspects of oral health, thus aiding routine clinical assessment. The objective of the study was to evaluate the oral-health related QoL in geriatric patients using the GOHAI.

## Materials and methods

Geriatric oral health assessment index

The GOHAI consists of 12 closed-ended questions (presented in Appendix I) evaluating self-perceived oral health, including pain, discomfort, and psychosocial impact [6]. The response to each question was assessed using a four-point scale, where 1 = never, 2 = sometimes, 3 = frequently, or 4 = always [7]. Responses scored as 1 and 2 were indicative of better oral health with no or few problems and good oral condition, whereas responses scored as 3 and 4 were indicative of poor oral health with multiple problems and bad oral condition. The study was conducted for a period of 2 months. The recruited subjects were divided into three age groups, those aged 65-69 years, those aged 70-74 years, and those aged 75 years or older.

The study was conducted in three stages. The first stage consisted of translating the questions on the GOHAI into Tamil, the local language, and the second stage consisted of its back translation into English. The third stage consisted of administering the GOHAI questionnaire to the study subjects. It was necessary to rephrase and shorten the questions on the GOHAI for more natural understanding. The administered form of the GOHAI questionnaire, modified to suit the target population, is shown in Appendix I.

Translation

Before administering the GOHAI to subjects with cultural and linguistic differences, it had to be translated and validated. Such translations pose two problems: 1) it may not be possible to translate some phrases or words directly and 2) languages exist within unique cultural and social frameworks, changing the meaning of some questions or making them meaningless. The GOHAI questionnaire was translated from English into Tamil by two dental students, who were fluent in both languages [8]. The Tamil version of the GOHAI questionnaire was then translated back into English by another dentist. The back-translated version and the original version were compared to determine if the questions had been adequately translated.

Participants

Subjects who visited the geriatric clinic of the Sri Ramachandra Institute of Higher Education and Research for three months were recruited. Subjects with cognitive and behavioral disorders and in-patients were excluded. The age, gender, and medical history of each participant were recorded, including whether subjects had diabetes mellitus or hypertension, whether they were smokers, and medicines taken. Dental histories were also recorded, such as denture usage, denture stomatitis, pigmentation of the tongue, and smokers’ palate.

Statistical analysis

All data were statistically analyzed using IBM SPSS Statistics for Windows, Version 24.0 (Armonk, NY: IBM Corp.). Chi-square test was performed for finding the significant differences between age groups.

The University Ethics Committee approved the protocol of this study (Ref: CSP/17MAY/58/159), and all enrolled subjects provided written informed consent before participating.

## Results

A total of 219 subjects aged 65 years or older were recruited. These included 126 (57.5%) subjects aged 65-69 years, 57 (26.0%) aged 70-74 years, and 36 (16.4%) aged 75 years or older. Table 1 shows their responses to the items on the GOHAI questionnaire, subdivided by subject age.

**Table 1:** Responses of subjects to questions on the Geriatric Oral Health Assessment Index by age

QUESTIONS	AGE IN YEARS	NEVER (1)	SOMETIMES (2)	FREQUENTLY (3)	ALWAYS (4)
Limitation of foods?	65-69	67.5%	31%	1.6%	0%
	70-74	19.3%	45.6%	29.8%	5.3%
	75 and older	0%	0%	36.1%	63.9%
Trouble biting firm meat and apples?	65-69	82.5%	17.5%	0%	0%
	70-74	5.5%	63.2%	31.6%	0%
	75 and older	5.6%	5.6%	38.9%	50.9%
Discomfort during swallowing?	65-69	78.6%	15.1%	4%	2.4%
	70-74	73.7%	12.3%	5.3%	8.8%
	75 and older	47.2%	22.2%	2.8%	27.8%
Prevents from speaking comfortably?	65-69	78.6%	13.5%	4%	4%
	70-74	66.7%	17.5%	8.8%	7%
	75 and older	58.35%	11.1%	5.6%	25%
Discomfort during eating?	65-69	38.1%	8.7%	3.2%	50%
	70-74	21.1%	10.5%	3.5%	64.9%
	75 and older	25%	13.9%	0%	61.1%
Contact with people limited by this condition?	65-69	74.6%	16.7%	5.6%	3.2%
	70-74	75.4%	15.8%	5.3%	3.5%
	75 and older	61.1%	13.9%	2.8%	22.2%
Unhappy with looks?	65-69	65.9%	20.6%	3.2%	10.3%
	70-74	61.4%	26.3%	7%	5.3%
	75 and older	50%	25%	2.8%	22.2%
Medication to relieve pain or discomfort?	65-69	58.7%	28.6%	5.6%	7.1%
	70-74	52.6%	28.1%	12.3%	7%
	75 and older	38.9%	38.9%	2.9%	19.4%
Worries about problems with teeth or denture?	65-69	27%	18.3%	31.7%	23%
	70-74	31.6%	57.9%	7%	3.5%
	75 and older	63.9%	30.65%	0%	5.6%
Felt nervous or self- conscious?	65-69	38.9%	7.1%	2.4%	51.6%
	70-74	38.6%	7%	5.3%	49.1%
	75 and older	25%	5.6%	0%	69.4%
Felt uncomfortable eating in front of other people?	65-69	74.6%	15.1%	4%	6.3%
	70-74	64.9%	26.3%	3.5%	5.3%
	75 and older	58.3%	19.4%	0%	22.2%
Sensitive to hot, cold or sweet foods?	65-69	50%	27.8%	7.9%	14.3%
	70-74	52.6%	21.1%	7%	19.3%
	75 and older	44.4%	22.2%	5.6%	27.8%

Responses to several GOHAI questions showed significant age-related differences. For example, 67.5% of subjects aged 65-69 years never experienced food limitations due to problems with teeth or dentures, whereas 63.9% of those aged ≥75 years always experienced these limitations; subjects aged 70-74 years showed intermediate responses (P = 0.00). Similarly, 82.5% of subjects aged 65-69 years never experienced difficulties biting firm foods, whereas about 50% of those aged ≥75 years always experienced difficulties (P = 0.00).

Discomfort during swallowing was never experienced by 78.6% of subjects aged 65-69 years, compared with 47.2% of those aged ≥75 years. Only 4% of subjects aged 65-69 years always experienced problems with speech, compared with 25% of subjects aged ≥75 years. The percentage limiting contact with other people due to problems with teeth or dentures was significantly lower in subjects aged 65-69 years than those aged ≥75 years (3.2% vs. 22.2%, P = 0.006). The percentages reporting that they never worried about problems with teeth or dentures was higher in subjects aged 65-69 years than those aged ≥75 years (27% vs. 63.9%), whereas the proportion reporting that they always felt uncomfortable eating in front of others was significantly lower in those aged 65-69 years than ≥75 years (6.35% vs. 22.2%, P = 0.024).

Responses to other questions on the GOHAI did not differ significantly among the three age groups.

## Discussion

The results of this cross-sectional study showed that older geriatric subjects aged 75 years or older perceived physical problems associated with dentition more acutely than psychological problems. The higher percentages of subjects aged ≥75 years than those in the other age groups experiencing food limitations and having trouble biting firm foods may have been due to differences in the percentages who wore dentures. Although the oldest age group had the highest percentage of edentulous subjects, it had the lowest percentage of denture wearers, 11.1%, compared with the groups aged 65-69 years (12.7%) and 70-74 (26.3%) years. Edentulous subjects have greater difficulty eating foods like raw carrots, apples, and nuts. [9]. Older age has been associated with a higher prevalence of edentulousness, which may correlate with the higher percentages of this group having food limitations and trouble biting [10].

Discomfort during swallowing was significantly more prevalent in subjects aged ≥75 years than in the other two groups. The prevalence of diabetes was higher in this group (30.6%) than in subjects aged 65-69 (29.4%) and 70-74 (28.1%) years. Diabetes has been associated with xerostomia, and xerostomia has been associated with swallowing difficulties [11-12].

Despite the age-associated increase in problems with dentition, the percentage of subjects who never worried about problems associated with teeth or dentures was higher in those aged 75 years or older (63.9%) than in those aged 65-69 years (27.0%) and 70-74 years (31.6%). The use of dental services is a consequence of the patient-perceived need for treatment [13]. Although two-thirds of elderly subjects have poor oral health, only one-third report concerns [14]. Subjects aged 60-64 years and 65-74 years were found to have a more favorable attitude towards dentistry than subjects aged 75 years or older [15]. Thus, despite having more dental problems, subjects in the oldest age group had fewer worries about problems related to teeth or dentures.

Speech problems due to problems with dentition were more acutely perceived by subjects aged 75 years or older than those aged 65-69 and 70-74 years. This difference may be due to the higher prevalence of edentulousness in the oldest age group [10]. Because the quality of speech production is significantly reduced after the loss of teeth, speech problems were perceived more by the oldest age group [16].

The percentages of subjects experiencing discomfort while eating were similar in the three age groups. More than 50% of subjects in each of these groups report always experiencing discomfort while swallowing, suggesting that this may be a common geriatric complaint, not specific to any particular age group. The percentage of subjects always limiting contact with others was higher in those aged 75 years or older than those aged 65-69 and 70-74 years. Edentate non-denture wearing subjects aged 75 years or older experience more trouble with speech formation than dentate individuals [16]. This difficulty with speech results in limiting contact with other people.

Responses to the other questions on the GOHAI did not differ significantly among the three age groups, suggesting that these problems may be perceived similarly across all geriatric age groups.

Limitations

The sample consisted mainly of women, preventing comparisons between men and women.

## Conclusions

This study revealed that perceptions of physical problems associated with oral health differed significantly among age groups in the geriatric population. The oldest group, those aged 75 years or older, reported physical problems more acutely than the other age groups. By contrast, problems related to appearance and attitude towards dental care were similar in the three age groups. Moreover, dental care was not regarded as a primary concern among geriatric subjects. Although these findings suggest that determination of the clinical and oral health of geriatric patients is required for optimum dental care, additional studies in larger populations are required to assess geriatric oral health.

## Appendices

Appendix I

GERIATRIC ORAL HEALTH ASSESSMENT INDEX QUESTIONNAIRE

1.        How often did you limit the kinds or amounts of food you eat because of problems with your teeth or dentures?

2.       How often did you have trouble biting or chewing different kinds of food, such as firm meat or apples?

3.       How often were you able to swallow comfortably?

4.       How often have your teeth or dentures prevented you from speaking the way you want?

5.       How often were you able to eat anything without feeling discomfort?

6.       How often did you limit contact with other people because of the condition of your teeth or dentures?

7.       How often were you pleased or happy with the looks of your teeth and gums or dentures?

8.       How often did you use medication to relieve pain or discomfort around your mouth?

9.       How often were you worried or concerned about problems with your teeth, gums or dentures?

10.      How often did you feel nervous or self- conscious because of problems with teeth, gums or dentures?

11.       How often did you feel uncomfortable eating in front of other people because of problems with your teeth or dentures?

12.      How often were your teeth or gums sensitive to hot, cold or sweet foods?

Response scale:

1 - never, 2 - sometimes, 3 - frequently, 4 - always
